# ePortfolios: Enhancing confidence in student radiographers' communication of radiographic anatomy and pathology. A cross‐sectional study

**DOI:** 10.1002/jmrs.787

**Published:** 2024-05-07

**Authors:** Magdalena Dolic, Yaxuan Peng, Keshav Dhingra, Kristal Lee, John McInerney

**Affiliations:** ^1^ Monash University Clayton Victoria Australia; ^2^ Royal Melbourne Hospital Parkville Victoria Australia

**Keywords:** eportfolios, image interpretation, radiographer commenting, radiography education delivery

## Abstract

**Introduction:**

In 2020, the Medical Radiation Practice Board of Australia made several revisions to its professional capabilities. To address this, medical radiation practitioners, including diagnostic radiographers, are required to escalate urgent findings in all radiographic settings. However, the confidence of radiographers in articulating descriptions of radiographic findings varies despite this requirement. This cross‐sectional study explores how the implementation of eportfolio affects student self‐perceived confidence in identifying and describing radiographic findings in both an academic and a clinical setting.

**Methods:**

A Qualtrics survey was distributed to second‐year radiography students who had used eportfolios. The survey comprised of four questions using a Likert‐scale and one open‐ended question. Quantitative data were analysed using the Wilcoxon signed‐rank test and qualitative data was thematically assessed.

**Results:**

Overall, 55 of 65 radiographic students (85%) completed the survey. Confidence (strongly agree and agree) decreased from 89% to 74% between academic and clinical environments when identifying abnormalities, and 89% to 73% when describing findings. This finding highlights the challenges students face when in the clinical environment. Wilcoxon signed rank test analysed a statistically significant relation between the two environments (*P* < 0.05). However, the relationship between identifying and describing skills was not statistically significant (*P* > 0.05). Following a review of the qualitative data, three recurring themes were identified among responses.

**Conclusion:**

ePortfolios assist in improving confidence in identification and description of radiographic abnormalities, particularly in an academic setting. The clinical environment presents unique challenges which may limit student clinical performance; however, this requires further investigation.

## Graphical abstract

This study investigates the impact of eportfolios on the confidence of second‐year radiography students in identifying and describing radiographic findings in academic and clinical settings. Results indicate a decrease in confidence in the clinical environment compared to the academic setting, highlighting challenges faced by students. While eportfolios show promise in enhancing confidence, further exploration of factors influencing clinical performance is warranted.

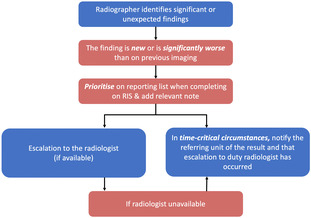

## Introduction

The Medical Radiation Practice Board of Australia (MRPBA) governs the practise of Australia's medical radiation practitioners to ensure safe practise of radiation for medical purposes.^1^ On 01 March 2020, the MRPBA released revised Professional capabilities for all medical radiation practitioners (MRP) in Australia. MRPs in Australia include radiation therapists, nuclear medicine technologists and diagnostic radiographers.^2^ Henceforth, we will refer to radiographers' role as this was the scope of the study.

**Figure 1:**
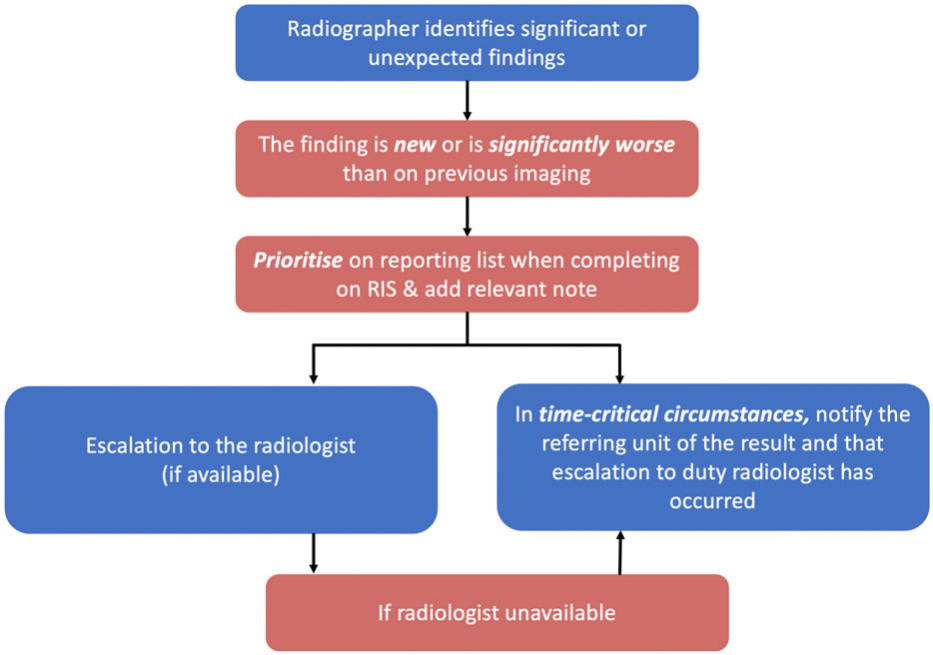
Flowchart depicting the process of escalation. RIS‐ Radiology Information System. This flowchart was developed by the Western Health Medical Imaging team to support staff in the escalation of urgent findings.

As part of the revised capabilities, Australian radiographers are required to not only evaluate their imaging for quality but also for the presence of any significant and/or unexpected findings across all radiographic settings (Fig. 1). And if detected, they must convey these findings in a timely manner to the appropriate persons for immediate patient management.^2^ This capability is supported by the policy entitled ‘Communicating Safely‐ if urgent or unexpected findings identified’.^3^ It has been published using the phrase ‘See something, Say something’ and also has been termed radiographer commenting or preliminary image evaluation.^4^ This is also a fundamental requirement of other regulatory bodies and professional organisations across the globe.^5^, ^6^ The change in policy in Australia reflects outcomes of coronial findings, whereby a failure to escalate findings resulted in delayed treatment and ultimately patient death.^7^ The escalation of urgent findings is also a fundamental requirement across several regulatory bodies and professional organisations across the globe; including but not limited to Australia and New Zealand.^8^, ^9^

A radiographer's evaluation is not a substitute for a radiology report.^10^, ^11^ A radiology report comprises formal documentation and communication regarding the results of a radiologic examination, performed by a radiologist.^10^ This differs from a radiographer's comment which focuses on timely communication and immediate management of urgent findings.^12^

Radiographer evaluation takes the form of verbal or written communication in relation to patient imaging.^10^, ^11^ This requires image interpretation skills, which comprises the integration of knowledge of radiographic anatomy and pathology to identify abnormalities including but not limited to fractures, dislocations and normal variants. Regardless of its format, unexpected or urgent radiographic findings must be communicated by appropriate means in a timely fashion.^2^, ^11^

Timely communication of urgent findings by radiographer boasts several benefits, including improvements in point‐of‐care patient management.^10^, ^11^, ^12^ Studies have shown that radiographer evaluation for significant findings can lead to improved clinical outcomes for patients, including prompt care and timely treatment, as well as diagnostic accuracy of acute abnormalities.^13^, ^14^, ^15^ However, barriers to radiographer commenting exist in forms of limited access to education opportunities, low confidence in interpreting images and busy workloads.^10^, ^11^, ^16^ Some studies have demonstrated that radiographers exhibit low confidence levels in communicating observations in appropriate medical terms.^10^, ^11^, ^16^ One solution that has shown promise is for radiographers to receive appropriate image interpretation education.^10^, ^17^, ^18^

In 2018, Monash University's Department of Medical Imaging and Radiation Sciences integrated eportfolios into its 4 years undergraduate radiographic studies. This programme aims to develop critically reflective radiographers with the multifaceted skill base to match.^19^ ePortfolios behave as an organised collection of learning evidence, foster long‐term professional growth, and encourage higher‐order critical thinking skills in learners.^20^, ^21^ eportfolios have benefits in higher education by promoting learning autonomy and accountability in students' learning.^20^, ^21^, ^22^, ^23^, ^24^, ^25^ At Monash University, eportfolios served as a formative assessment tool during the first semester of first year, specifically integrated into anatomical studies. The eportfolios consisted of 3D computed tomography data sets, images of bones and radiographic images provided by the department, to assist in building the eportfolio. More specifically, these activities required students to annotate and describe findings observed on images provided to demonstrate an understanding of relevant anatomy and pathology. The final formative assessment required students to present case studies. This was based around an annotated radiograph and required the appropriate use of medical terminology (i.e. angulation, location, type of fracture) to describe the radiographic findings. This gave students an opportunity to develop their image interpretation skills and establish confidence when implementing medical terminology to describe radiographic findings. Ultimately, students were responsible for curating artefacts evidencing their learning of anatomy and pathology. Students were also provided with regular feedback from academic supervisors regarding their progress and support was available in the form of weekly tutorials.

The use of eportfolios has been implemented over the past decade in healthcare professions spanning medicine, nursing, midwifery, radiation therapy and dietetics internationally.^26^, ^27^, ^28^, ^29^, ^30^ They have been used to assist students and educators to undertake formative assessment, monitor progress and set goals.^26^, ^27^, ^30^ Beyond education, other studies have found benefits of eportfolios as a tool for tracking professional development and forming links between academic and clinical practice.^28^, ^29^ No literature to the authors' knowledge which explores whether a correlation exists between eportfolios and confidence in communicating radiographic anatomy and pathology currently exists.

This study aimed to explore how the implementation of eportfolios affects student confidence in identifying and describing radiographic findings in both an academic and a clinical setting.

## Methods

### Study design

The study employed a cross‐sectional survey design where both quantitative and qualitative data were collected and analysed concurrently to address the aim.

### Ethics approval

The ethics approval of this project (ID 19122) was granted in October 2019 by the Monash University Human Research Ethics Committee.

### Data collection

In October 2019, online responses were collected from second‐year Monash University radiography students who had completed an anatomy and pathology eportfolio assessment in year one. The survey utilised the Qualtrics™ online tool and employed clear language in formulating quantitative and qualitative questions. To ensure clarity, the survey was pilot‐tested on an academic staff member, and participants were specifically second‐year radiography students at Monash University who had curated an anatomy and pathology eportfolio as an assessment piece in year one of their degrees.

Researchers YP and MD presented the research project's explanatory statement using PowerPoint slides during the final 10 min of a tutorial. Importantly, both researchers were not involved in teaching or assessing students, eliminating potential bias in participant responses. The explanatory statement, including a survey link, was distributed to potential participants via the Learning Management System, Moodle.

The survey consisted of two sections. The first section involved four quantitative statements (Table 1). Participants indicated their level of agreement/disagreement with the statement using a Likert scale of four options with ‘1’ being strongly disagree and ‘4’ being strongly agree. The first and second statements assessed whether eportfolios assisted students in confidently identifying radiographic pathology. Statement one focussed on an academic context, that is, in a university setting, during lectures, tutorials and assessments. The second statement focused on the clinical context, that is, during clinical placement. The third and fourth statements assessed whether eportfolios assisted students in confidently describing radiographic pathology. The third statement examined the academic context whereas the fourth one focused on the clinical context. The keywords, ‘identifying’, ‘describing’, ‘at university’ and ‘during clinical placement’, were emphasised in bold in the survey to draw students' attention to the keywords and prevent confusion from similar question structures. The second section of the survey was a single open‐ended question, ‘Were there any complications in clinical placement compared to university that prevents you from confidently commenting on the radiographic pathology’? Students were provided with a textbox to express their perspectives.

**Table 1:** Qualtrics™ survey statements.

	Likert Statements
Statement 1	Creating eportfolios in the first year enhanced my confidence in **identifying** radiographic pathology **at university**. That is, during discussion with peers in lectures and tutorial.
Statement 2	Creating eportfolios in the first year enhanced my confidence in **identifying** radiographic pathology **during clinical placement**.
Statement 3	Creating eportfolios in the first year enhanced my confidence in **describing** radiographic pathology **at university**.
Statement 4	Creating eportfolios in the first year enhanced my confidence in **describing** radiographic pathology **during clinical placement**.

### Data analysis

Likert scale data were analysed using descriptive statistics by reporting percentages in Microsoft® Excel® (2019). To demonstrate any statistical relationship between students' perceptions, a Wilcoxon signed‐rank test performed using IBM SPSS statistics for Windows 24.0 (IBM Corp.: Armonk, NY, USA) was employed due to the non‐parametric yet paired nature of the data.^31^ The significance level was defined as *P* < 0.05.

Braun and Clarke's method for thematic analysis was chosen as the framework for analysis of the qualitative data. It was employed as it presents a flexible and robust method of analysis and is well accepted in the health sciences.^32^ YP and MD completed the analysis with guidance provided by JM, who had significant experience conducting thematic analyses. The six‐step approach allows for the identification of patterns, referred to as themes, in data (Table 2). The iterative nature of the framework means that a review of the importance of the themes is conducted.^32^

**Table 2:** Table depicting how qualitative responses were coded and allocated to themes, focusing on excerpts under the theme ‘Pressure associated with clinical placement’.

Stage		Process
1	Familiarisation	Data transcribed from Qualtrics™ All data were read on first review with initial ideas noted. (YP, JM and MD). E.g. ‘…perhaps there was more pressure to get the answer correct in clinical placement than at uni’, ‘a lot more complex cases’ and ‘This is not enough for detail describing the pathologies like fracture type ect [Sic]’.
2	Coding	Data systematically collated into codes. E.g. Time constraints, Judgemental environment, student credibility and differences between tutor and student. (YP and MD)
3	Theme development	Codes were then collated into potential themes. E.g. Pressure. (YP and MD)
4	Refinement	Authors confirmed the relevance of derived themes to the entire data set. (YP, MD and JM)
5	Naming	Further analysis of codes to attain a clear understanding and title for each theme. For example, title evolved from ‘Pressure’ to ‘Pressures associated with clinical placement’. (YP, MD and JM)
6	Writing up	Themes were analysed with reference to the objective, initial question and potential interlinks between themes. (YP, MD, KD and JM)

## Results

**Figure 2:**
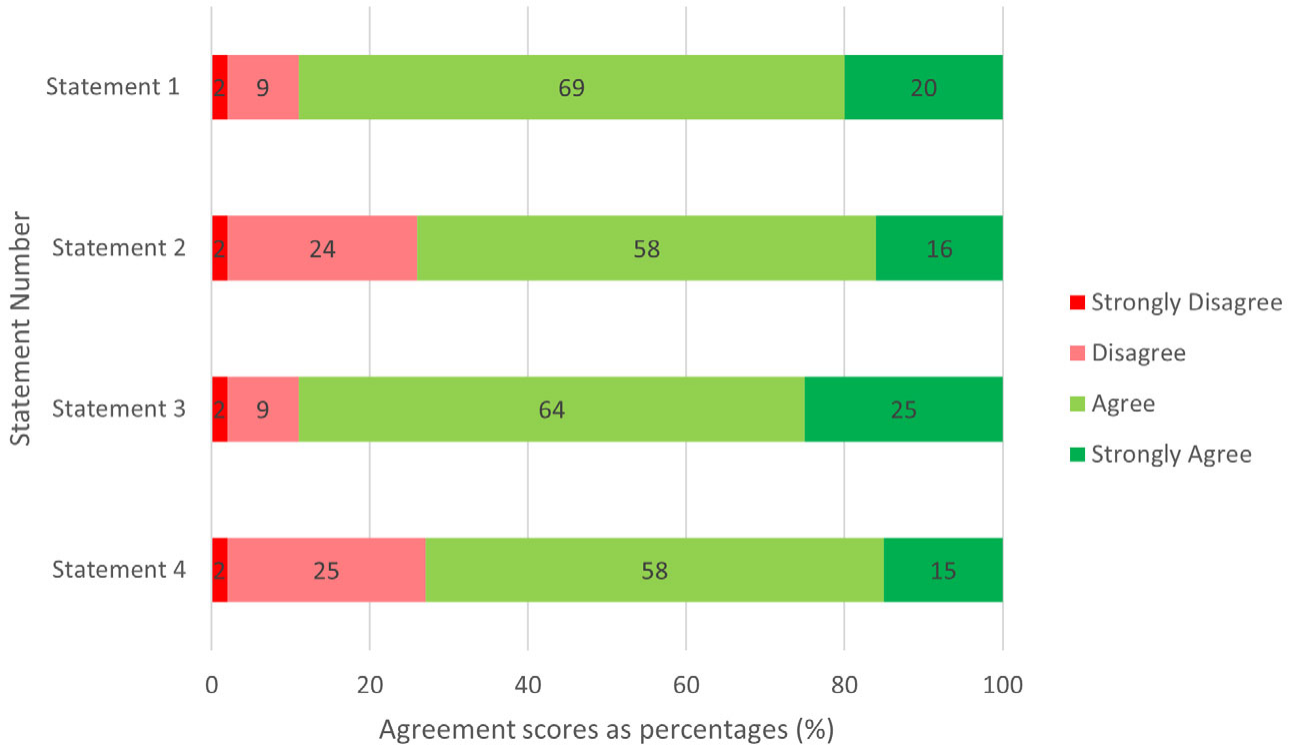
Likert responses presented as percentages.

Overall, 55 of 65 radiographic students (85%) completed the survey. Responses to the Likert statements were overall positive, with the majority of students agreeing that the eportfolios improved their confidence (Fig. 2).

Forty‐nine students (89%) agreed or strongly agreed that eportfolios enhanced their confidence in identifying radiographic pathology at university. Comparatively, 41 (74%) students agreed or strongly agreed that eportfolios enhanced their confidence in identifying radiographic pathology in the clinical setting. A similar trend was shown in describing radiographic pathology with a decrease of overall agreement from 49 students (89%) in the academic setting to 40 students (73%) in the clinical setting. These trends are shown in Table 3. These differences between the students' perceived confidence in the academic compared to the clinical settings for identifying and describing radiographic pathology proved to be statistically significant (*P* = 0.012 and *P* = 0.002, respectively). When comparing the paired groups between confidence in identifying and describing, there was no significant difference in a university (*P* = 0.513) or a clinical (*P* = 0.638) setting (see Table 4).

**Table 3:** Students' self‐perceived confidence in using eportfolios.

	Statement 1	Statement 2	Statement 3	Statement 4
Strongly agree & agree	89%	74%	89%	73%
Strongly disagree & disagree	11%	26%	11%	27%

**Table 4:** Relationship between Likert statements using the Wilcoxon signed‐rank test (* denotes significance).

	*P*‐value
Relationship between students' confidence in **identifying** radiographic pathology in an academic environment and a clinical environment (statements 1 and 2)	0.012*
Relationship between students' confidence in **describing** radiographic pathology in an academic environment and a clinical environment (statements 3 and 4)	0.002*
Relationship between identifying and describing radiographic pathology in an **academic** environment (statements 1 and 3)	0.513
Relationship between identifying and describing radiographic pathology in a **clinical** environment (statements 2 and 4)	0.683

A thematic analysis of the qualitative data using the Braun and Clarke^29^ method highlighted three main challenges to the communication of radiographic pathology within a clinical environment. These themes were:

1. Pressures associated with clinical placement.
2. A greater variety of subtle pathologies and normal variants seen on placement.
3. Poor consolidation of knowledge at university.

Of those who completed the survey, 38 (69%) provided responses to the qualitative question, with one student providing an invalid response, ‘//’ (Respondent 18). Only six students felt that they did not encounter specific challenges during clinical placement. Thirty‐one students documented challenges specific to clinical placement which hindered their confidence in communicating radiographic pathology.

### Theme 1: pressure associated with clinical placement

Pressure associated with clinical placement was the strongest theme among participants. Twenty‐one (55%) of respondents felt that it could undermine students' confidence in communicating radiographic pathology.

Several respondents acknowledged anxiety and fear when interpreting radiographs in front of qualified radiographers during their clinical placements.

Worried about getting the answer wrong in front of people they didn't know well (Respondent 46).

There were additional pressures in clinical sites due to the presence of professionals overlooking your evaluation of an image which made it easy to second guess yourself (Respondent 44).

Seeming foolish in front of supervisors/experienced radiographers (Respondent 53)

Respondents also reported that *‘clinical placement [was] more stressful and more difficult’* than the academic environment (Respondent 34). Other responses cited reasons such as being *‘scared of giving the wrong answer’* (Respondent 35) and *‘time constraints’* (Respondents 8, 9, 22, 27, 49, 52). In comparison, *‘University is usually more forgiving’* (Respondent 27) allowing students to *‘have a go/educated guess’* (Respondent 30).

### Theme 2: a greater variety of subtle pathologies and normal variants seen on placement

The large variety and subtle differences between pathologies were brought up as a barrier to confidence in commenting on clinical placement.

Just being able to clearly distinguish between two very similar forms of pathology (Respondent 15).

Pathology seen in clinical placement might be subtle, less easy to be picked up compared to obvious pathologies seen in lecture slides (Respondent 36).

Clinical setting is more serious; the pathology is a lot more subtle than when we study them in the classroom (Respondent 40).

Several respondents attributed variations in anatomy and pathology as a unique challenge in the clinical environment.

Abnormal variations in pathology (Respondent 21).

Terminology at sites could vary from that in which we learnt; variances were minor but still evident (Respondent 24).

Variation in patient anatomy (Respondent 51).

### Theme 3: poor consolidation of knowledge at university

Multiple factors, including intrinsic study habits and content coverage, were identified by students as barriers to confidently identifying and describing radiographic pathology. One participant commented on *‘not actively revising…after university to solidify the content’* (Respondent 13). However, the biggest issue identified by students within this theme was the limited extent to which anatomical and pathological concepts were covered at University.

This is not enough for detail describing the pathologies like fracture type (Respondent 29).

Main reason being that we were not able to go into depth in regard to chest pathology in a e‐portfolio aspect (Respondent 31).

There were limited radiographic images that we used; I feel like we more learnt about the anatomy rather than radiographic pathology (Respondent 33).

The vast variety of normal variants which were not discussed in a lot of detail at university (Respondent 48).

Sometimes I was not completely sure if what I identified was correct, as we may not have been taught about at uni (Respondent 41).

Different teaching styles between academic and clinical education may also have impacted students' confidence as *‘the way clinical tutors [teach may be] different from the eportfolio’* (Respondent 37).

## Discussion

To meet accreditation requirements, Universities in Australia must integrate learning programs which ensure students demonstrate key professional capabilities prior to qualification.^33^ To achieve this, educators formulate programs that strive to deliver innovative teaching practices to influence clinical practice. ePortfolios offer one approach to facilitate students' learning and ensure they have the required evidence upon graduation. This study offers a unique snapshot into the role eportfolios play in enhancing student self‐perceived confidence when articulating radiographic findings, a key requirement for MRPs comprising Domain 1 key capability 7 (See excerpt below).

1. Evaluate radiological images for quality and identification of any urgent and/or unexpected findings;
2. Where urgent and/or unexpected findings are identified, take appropriate and timely action to ensure the immediate management of the patient.^1^

Taking appropriate and timely action is a key responsibility if a radiographer identifies radiographically significant findings on an image and must be interpreted.^2^, ^34^ That is, as registered healthcare workers, radiographers must ensure information relating to patient safety is clearly communicated to the appropriate persons ‘who may include the reporting medical specialist, the requesting practitioner or other practitioners, for the immediate and appropriate management of the patient/client’ (p.9).^2^

Image interpretation education exists in various forms and is accessible in undergraduate and postgraduate programmes.^14^ This includes short courses, intensive training programmes and post‐graduate master's degrees.^10^, ^13^, ^15^, ^17^, ^18^ Radiographer image interpretation education can be challenging. Knowledge retention and the time commitment required to complete such programmes of learning an approach to long‐term and self‐reflective implementation.^15^, ^17^ ePortfolios can complement existing programmes of learning as an approach to long‐term and self‐reflective learning.^23^, ^29^ ePortfolios have progressively grown in popularity and have been shown to develop students' professional skills.^20^, ^35^, ^36^, ^37^ While their effectiveness has been investigated in healthcare professions such as medicine and nursing,^26^, ^27^, ^28^, ^29^, ^30^ currently there is limited research to the authors' knowledge relating eportfolio's use with the communication of significant radiographic findings by radiography students.

Early studies show that radiographers exhibit low confidence in image interpretation, with more recent studies demonstrating similar findings.^10^, ^11^, ^13^, ^14^ Smith et al. also found that ‘radiographers [had] difficulty converting their observations into words’.(p.7)^18^ This study has demonstrated positive results regarding students' perceived confidence, in academic and clinical environments, when identifying and describing radiographs. The majority of students agreed that eportfolios improved their confidence. This positive outcome might be attributed to several reasons. These reasons include the value of an eportfolio as a learning resource that students can utilise to monitor and reflect on their learning.^17^, ^20^, ^21^, ^23^, ^25^, ^29^ However, the structure and content of eportfolios must be tailored to the specific learning outcomes.^27^, ^30^

When comparing the paired groups between identifying and describing, there was no statistically significant difference between the two settings. This differs somewhat from what is reflected in the literature, whereby radiographers are less confident in their ability to describe findings than their ability to identify findings.^10^, ^11^, ^16^ ePortfolios, however, allow students to work in a group and present radiographic cases using appropriate terminology. Through this formative assessment, eportfolios may aid in the expansion of their describing capacity.

Students expressed an overall positive sentiment to the use of eportfolios in both academic and clinical environments. A majority of students agreed or strongly agreed that ePortfolio activities, conducted at university, enhanced their confidence when describing within a clinical setting. This strongly suggests that eportfolios, when used as an educational tool at university, have the potential to enhance students' confidence in a clinical setting. Furthermore, this implies that eportfolios have the capacity to facilitate transferability of learning between settings. However, the significant reduction in overall confidence between the academic and clinical environments cannot be overlooked. The qualitative data provided important insight into why this difference occurred. Theme one revealed that a large number of students attributed this to pressures associated with the clinical environment that were not present in the university setting. Students find it challenging in clinical settings when encountering challenges such as time pressure and fear of making mistakes.^38^, ^39^, ^40^ A recurring theme among the participants was the presence of a ‘supervisor’ overlooking their work. Trying to meet supervisors' expectations while focusing on patient care, students are likely to ‘second guess’ (respondent 44) their opinions and perform with lower confidence.^40^, ^41^

Theme two identified the unique challenge of recognising subtle pathologies and the extensive number of normal variants encountered within a clinical setting. In contrast, theme three focused on the academic environment where limited knowledge and poor consolidation of content were identified as a barrier to transition of learning into clinical practice. When themes two and three are reviewed in conjunction with one another, there appears to be a discrepancy between students' perceived expectations and what was required. The MRPBA clearly highlights that it is urgent and/or unexpected findings that radiographers need to identify and communicate.^2^ These urgent findings include but are not limited to acute dislocations, fractures, pneumothorax and mis‐positions of tubes/lines.^42^ This mirrors the expectations set by other professional and regulatory bodies across the globe.^8^, ^9^, ^43^ This highlights that a discrepancy may exist between the required and self‐perceived expectations within the clinical environment.^39^ To address this discrepancy, students should be informed clearly of the expectations while on placement as the scope of radiographic pathology is impossible to cover during academic curricula. These expectations should be formulated around the registration requirements of each individual countries' regulatory body so that students can perform competently in the clinical environment.^2^, ^5^, ^6^ In addition, to ensure the successful transferability of eportfolio benefits, key inhibitors to student confidence should be addressed prior to the commencement of clinical placement.

### Limitations

Limitations of the study should be considered when interpreting this study's data. Firstly, these results were from a single institution with a sample size of 55 participants. The authors recognise that the study sample size may not be representative of all student radiographers using eportfolios, thus may have influenced the statistical analysis performed. Secondly, this study investigated students' perceived confidence rather than measurable outcomes such as test results to assess students' performance. Lastly, this survey was distributed after an extended period following the conclusion of first year placements. As participants undertook the survey in second year, their perceptions towards eportfolios and their ability to interpret images confidently may have been different from their first‐year perceptions, hence raising the possibility of recall bias.

## Conclusion and future recommendations

ePortfolios enhanced students' perceived confidence in identifying and describing radiographic pathologies in both academic and clinical environments. Influencing factors for this included the longevity of eportfolios as a learning tool and the enhancement of students' creative and reflective skills. Notably, there was no statistically significant difference between identifying and describing confidence. This suggests similar confidence in describing capacity possibly due to the novelty of eportfolios and their integration as formative assessments as opposed to previous literature.

A reduction of perceived student confidence was identified in the transition between the academic to the clinical environment. Pressure associated with the clinical environment was identified as the largest contributing factor. Other factors included pathological/anatomical variability and a lack of university‐based comprehension of content. These inhibitors should be addressed before clinical placements to maximise successful transferability of eportfolio benefits in two environments.

Future research should focus on a study design that assesses eportfolio learning on a longitudinal scale. Incorporating second to fourth year students, as well as qualified radiographers would provide this scope to assess eportfolio learning on a longitudinal scale to measure growth.

## Conflict of Interest statement

The authors declare no conflict of interest.

## Data Availability

The data that support the findings of this study are available from the corresponding author upon reasonable request.
